# A novel homozygous *TUB* mutation associated with autosomal recessive retinitis pigmentosa in a consanguineous Chinese family

**DOI:** 10.1186/s12920-023-01430-0

**Published:** 2023-01-18

**Authors:** Wei Xu, Ming Xu, Qinqin Yin, Chuangyi Liu, Qiuxiang Cao, Yun Deng, Sulai Liu, Guiyun He

**Affiliations:** 1 Central Laboratory, Hunan Provincial People’s Hospital (the First Affiliated Hospital of Hunan Normal University), Changsha, 410000 China; 2 School of Medicine, Changsha Medical University, Changsha, 410219 China; 3 Department of Ophthalmology, Hunan Provincial People’s Hospital (the First Affiliated Hospital of Hunan Normal University), Changsha, 410000 China; 4 School of Life Sciences, Hunan Normal University, Changsha, 410081 China

**Keywords:** Retinitis pigmentosa, Retinal dystrophy, WES, *TUB*, Consanguineous family

## Abstract

**Background:**

Retinitis pigmentosa (RP) is the most common type of inherited retinopathy. At least 69 genes for RP have been identified. A significant proportion of RP, however, remains genetically unsolved. In this study, the genetic basis of a Chinese consanguineous family with presumed autosomal recessive retinitis pigmentosa (arRP) was investigated.

**Methods:**

Overall ophthalmic examinations, including funduscopy, decimal best-corrected visual acuity, axial length and electroretinography (ERG) were performed for the family. Genomic DNA from peripheral blood of the proband was subjected to whole exome sequencing. In silico predictions, structural modelling, and minigene assays were conducted to evaluate the pathogenicity of the variant.

**Results:**

A novel homozygous variant (NM_003320.4: c.1379A > G) in the *TUB* gene was identified as a candidate pathogenic variant in this parental consanguineous pedigree. This variant co-segregated with the disease in this pedigree and was absent in 118 ethnically matched healthy controls. It’s an extremely rare variant that is neither deposited in population databases (1000 Genomes, ExAC, GnomAD, or Exome Variant Server) nor reported in the literature. Phylogenetic analysis indicated that the Asn residue at codon 460 of TUB is highly conserved across diverse species from tropicalis to humans. It was also completely conserved among the TUB, TULP1, TULP2, and TULP3 family proteins. Multiple bioinformatic algorithms predicted that this variant was deleterious.

**Conclusions:**

A novel missense variant in *TUB* was identified, which was probably the pathogenic basis for arRP in this consanguineous family. This is the first report of a homozygous missense variant in *TUB* for RP.

**Supplementary Information:**

The online version contains supplementary material available at 10.1186/s12920-023-01430-0.

## Introduction

Retinitis pigmentosa (RP), a highly heterogenous genetic disorder, is the most common inherited retinopathy induced by progressive loss of photoreceptor cells and subsequent degeneration of the retina. Typical clinical manifestations of RP include night blindness, bone-spicule deposits, gradual loss of central vision, waxy optic disc, and attenuated retinal arteries. The prevalence of RP varied significantly among ethnic groups ranging from 1/1000 to 1/ 5000 worldwide [1–3]. RP can be inherited in classic Mendelian inheritance (including 20–25% autosomal recessive, 15–20% autosomal dominant, and 10–15% X-linked) [2] as well as rarely mitochondrial [4] and digenic [5] inheritance. Although most cases of RP are monogenic, the disease is highly genetically heterogeneous. To date, at least 69 genes have been identified for nonsyndromic RP (including 23 genes for adRP, 44 genes for arRP, and 2 for xlRP) (RetNet, https://sph.uth.edu/retnet, updated October 7, 2022). Generally, broad functions of the identified genes can be classified into five groups: RNA splicing, cellular structure, retinal metabolism, photo-transduction, and tissue development and maintenance [6]. These genes, however, account for no more than 60% of all patients [7, 8], and the rest remain genetically unsolved. In China, mutations in about 34 genes have been detected, among which the top seven genes are *CYP4V2*, *RHO*, *USH2A*, *RPGR*, *CRB1*, *RP2*, and *CHM*, accounting for about two thirds of the RP-related mutations in our Chinese population [9]. Identification of additional genes or novel variants is a prerequisite for genetic counseling, and it’s key to better define the genetic etiology of RP. The remarkable genetic and phenotypic heterogeneity of arRP, however, remains a major obstacle in the identification of pathogenic variants. In addition, some homozygous variants are too rare to be screened in general case–control studies. Whereas pedigrees with hereditary diseases, especially consanguineous pedigrees, are extremely valuable resources for genetic studies of inherited diseases such as arRP.

*TUB* (MIM #601197), also known as *rd5* or *RDOB*, maps to 11p15.4 and encodes the tubby-bipartite transcription regulator [10, 11]. Abundantly expressed in the retina, brain, and cochlea [12–14] with dual localization to the plasma membrane and nucleus [10], *TUB* has been implicated in energy balance [15, 16], retinal degeneration [12], and neuronal regulation in mice [17]. However, the underlying molecular mechanism remains to be fully elucidated. Previous studies have demonstrated that loss-of-function mutations in *Tub* or Drosophila king tubby *(ktub)* cause retinal degeneration in both mice and Drosophila [18–21]*.* Mutations in *TUB* for retinal-associated diseases in humans, however, are extremely rare. Thus far, only one mutation in *TUB* for arRP have been reported in the literature, in which a homozygous frameshift variant resulted in retinal dystrophy, hearing loss, and obesity in a consanguineous UK Caucasian family [12]. Here, we describe a novel rare homozygous *TUB* variant (NM_003320.4, c.1379A > G, p.Asn460Ser) in a Chinese consanguineous family with typical arRP. In silico predictions, structural modelling, and experimental studies were conducted to evaluate the pathogenicity of this variant.

## Materials and methods

### Participants and clinical assessment

**Fig. 1:**
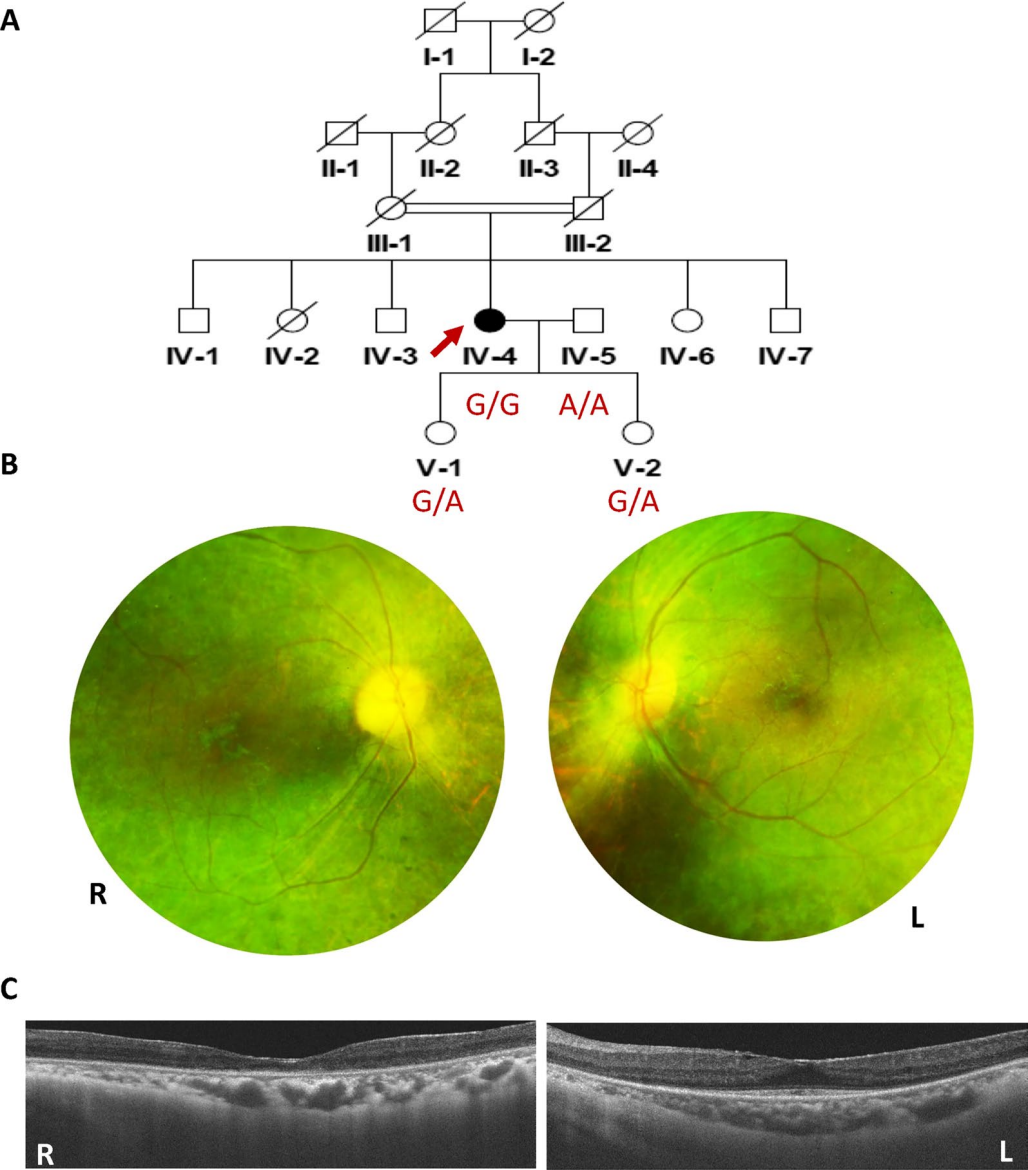
Pedigree of the arRP family, Fundus and OCT images of the proband. **A** Pedigree of the parental consanguineous Chinese family with RP (red arrow indicates the proband of this pedigree, slashed symbols represent deceased members). **B** Fundus images of the proband’s two eyes (R: right, L: left). **C** Optical coherence tomography (OCT) images of the proband (R: right, L: left)

A Chinese parental consanguineous family (living in the remote countryside of Hunan Province) with arRP (Fig. 1) and 118 ethnicity and region-matched healthy adults were enrolled by the Department of Ophthalmology of Hunan Provincial People’s Hospital. Overall ophthalmic examinations including funduscopy, decimal best-corrected visual acuity (BCVA), axial length and electroretinography (ERG), were performed by senior ophthalmologists. This study was approved by the Ethics Committee of Hunan Normal University.

### WES analysis

Genomic DNA from peripheral blood leukocytes of the proband was subjected to WES analysis on Illumina NovaSeq 6000 at the Berry Genomics as described previously [22]. Briefly, data were aligned to hg38/GRCh38 with BWA and the average coverage depth was about 100×. The VeritaTrekker® Variant Detection System, Enliven® Data Annotation, and Interpretation System were used to perform SNP, CNV, indel discovery, and genotyping. All nonsynonymous variants (NSVs) including single nucleotide variants (SNVs), splicing sites, and indels were selected and filtered against the dbSNP137 database, the ExAC, gnomAD, 1000 Genomes phase3, and the MyGenostics local database. The variants with a minor allele frequency (MAF) less than 1% were kept as candidates. Pathogenicity of the mutants were assessed following the guidelines of the American College of Medical Genetics and Genomics (ACMG) [23, 24]. Benign or Likely-benign mutations were filtered out.

### Validation and segregation analysis of candidate mutations, and in silico analysis

Primers (Additional file 1: Table S1) used to amplify the fragments harboring candidate variants were designed using Primer3 (https://bioinfo.ut.ee/primer3-0.4.0). Samples of 118 ethnicity and region matched healthy controls were also sequenced to screen variants in *TUB.* SIFT (Sorting Intolerant from Tolerant, http://sift.jcvi.org), Mutation Taster (http://www.mutationtaster.org), PROVEAN (Protein Variation Effect Analyzer, http://provean.jcvi.org), and CADD_Phre (Combined Annotation Dependent Depletion, http://cadd.gs.washington.edu) were used to evaluate the potential pathogenicity of the variant with default parameters. Novopro (https://www.novopro.cn/tools/protein-hydrophilicity-plot.html) was used to evaluate the hydrophobicity of the mutant TUB protein. The sequence alignment of different species were performed using ClustalW (https://www.ebi.ac.uk/Tools/msa/clustalo). The three dimensional protein model was generated with PyMOL (http://www.schrodinger.com/pymol/) [25].

### Minigene splicing assay

The 2240-bp DNA fragments of *TUB* (including exons 10–12 and introns 10–11) were amplified from the genomic DNA of the proband or a healthy control with primers as the following: *TUB*-BamHI-F: 5′- aagcttggtaccgagctcggatccGTCCAACTTGATGGGCACCAAGTTCACT-3′; *TUB*-XhoI-R: 5′- ttaaacgggccctctagactcgagGGTCATTGCCATGGATGATCTGGAAGTT-3′. After treatment with restriction endonucleases BamHI and XhoI, the amplified fragments were cloned into pMini-CopGFP using the KOD-Plus Neo Kit (TOYOBO CO., OSAKA, JAPAN). The recombinant constructs were confirmed by Sanger sequencing. The wild type (pMini-CopGFP-*TUB-*WT) and mutant type (pMini-CopGFP-*TUB-*MT) recombinant constructs were then transiently transfected into two commonly used mammalian cell lines (APRE-19 and 293 T cells) following the protocol provided by the TurboFect kit (Thermo, USA). RT-PCR was performed to obtain the target fragments of human *TUB* gene with primers as the following: MiniRT-F: 5’-GGCTAACTAGAGAACCCACTGCTTA-3’; *TUB*-RT-R: 5’-GGTCATTGCCATGGATGATCTG-3’. The amplified fragments were detected on 1.5% agarose gel and further verified by Sanger sequencing.

## Results

### Clinical evaluation

In this five-generation parental consanguineous Chinese pedigree (Fig. 1A), the proband (IV:4) and three healthy individuals (IV:5, V: 1, and V: 2) were enrolled in this study. Comprehensive ophthalmic examinations were performed for them. The proband exhibited night blindness and decreased visual acuity since the age of 16, and received a well-defined clinical diagnosis of RP at age 35. The last examination was carried out at age 59 with a BCVA of index/20 cm and 0.25 in the right and left eyes. The axial length was 22.40 mm in the right eye and 23.60 mm in the left eye. Funduscopy displayed spread attenuated arterioles, waxy optic disc, tunnel field, diffused bone-spicule deposition, and atrophy of the choroid in both eyes (Fig. 1B). OCT images showed perifoveal retinal thinning in both fundi (Fig. 1C). At ages 53 and 59, the proband underwent cataract surgery in the right eye and left eye, respectively. In addition, the proband exhibited no other related disorders nor additional phenotypes such as obesity or hearing deficits as described in other report [12]. The husband and two daughters of the proband were asymptomatic with normal funduscopic observations.

### WES analysis and variants validation

WES was performed for the proband. After mapping to the human reference genome (hg38), we achieved 3.2G bases with a mean sequencing depth of 100×, among which ≥ 20 × read depth were approximately 98.92%. A total of 53,556 genetic variants including indels and SNPs were detected in the initial WES data of the proband. After exclusion of high-frequency variants in th**e** gnomAD, 1000G phase III, ExAC, and MyGenostics local database, four candidate variants including three heterozygous variants in *RAX2, RP1,* and *PITPNM3,* and a homozygous variant in *TUB*, were remained as suspected pathogenic variants (Additional file 1: Table S2). Further co-segregation investigation and genotype–phenotype correlation analysis excluded the pathogenicity of the three heterozygous variants in *RAX2, RP1* and *PITPNM3.* A novel homozygous mutation in *TUB* (c.1379A > G. p.Asn460Ser) remained as the only candidate variant, and no additional causative mutations in all other known RP genes were detected.

**Fig. 2:**
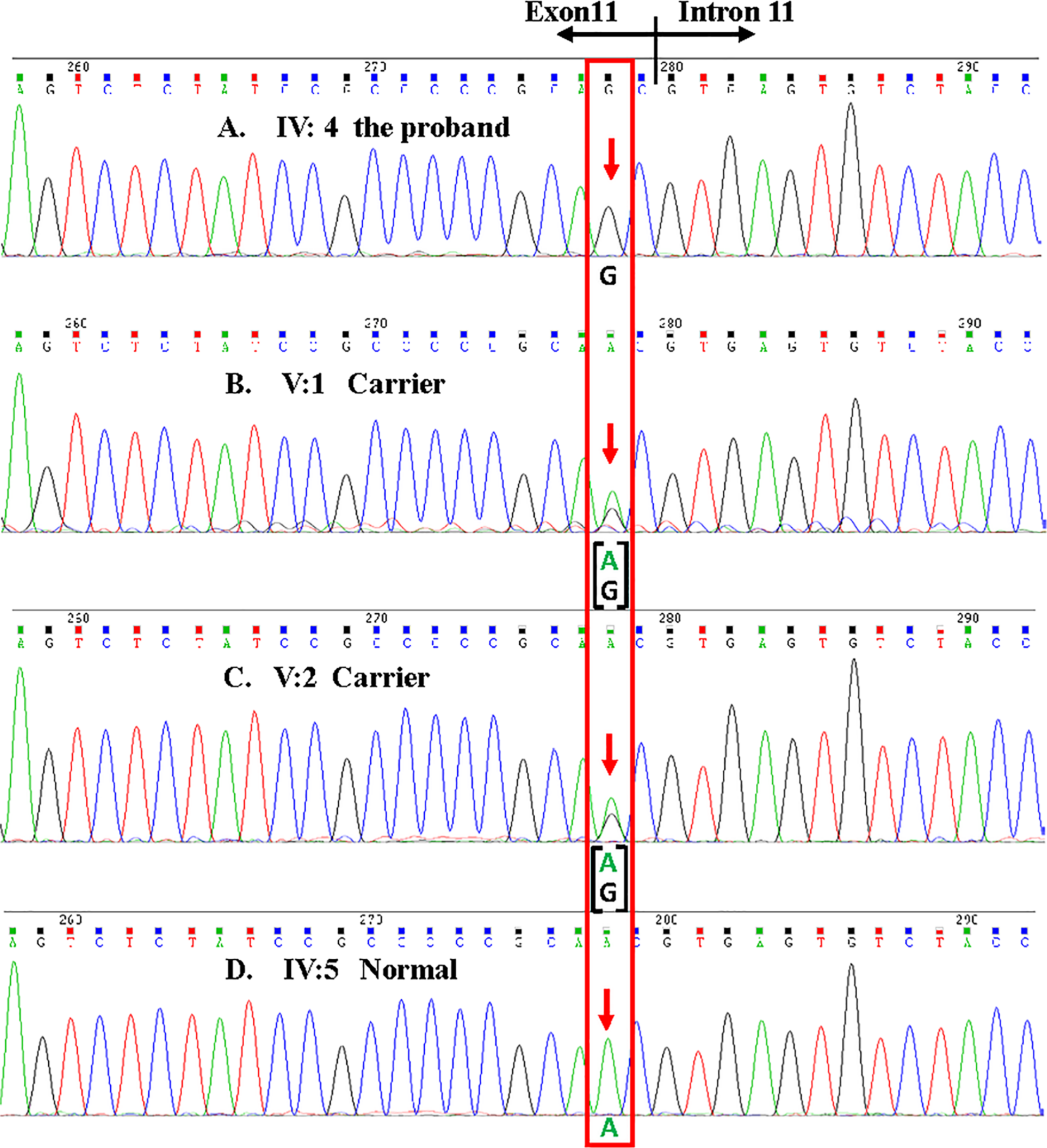
Co-segragation analysis of the c.1379A > G (p.Asn460SerSer) variant in *TUB.* **A** Sequences of the homozygous c.1379A > G variant in the proband (IV:4); **B**, **C** Sequences of the heterozygous carries (V:1, V:2) of the family; **D** Sequences of healthy member (IV:5) of the family

Further Sanger sequencing validated the proband (IV: 4) harbored the *TUB* variant in a homozygous state, whereas his husband (IV: 5) was homozygous for the wild type alleles and two unaffected daughters (V: 1, V:2) were heterozygous carriers for the variant (Fig. 2). The homozygous variant, based on Sanger sequencing, was verified to co-segregate with the RP phenotype in an autosomal recessive inheritance manner and was excluded from 118 ethnically and regionally matched normal controls. Although the same variant deposited in ClinVar (variation ID: 1,091,773) was interpreted as “likely benign”, we propose that such “likely benign” interpretation is invalid since detailed information about the variant including the variant type, the inheritance pattern, the ethnic group, affected status, or allele origin were not provided. Furthermore, the same variant was also deposited in dbSNP as a SNP (rs189328202) with a frequency of 0.00080, 0.000159 and 0.00005 in 1000G, ExAC, and GnomAD, respectively (Table 1). Importantly, the variant was not reported in a homozygous state in all public database (Table 1). This novel homozygous variant (c.1379A > G. p.Asn460Ser) in *TUB* appeared to be extremely rare as it was not reported in the literature either.

**Table 1:** Allele frequency (G) of c.1379A > G in exon 11 of *TUB* (rs189328202) among different population (Released April 9, 2022)

	Number of Homozygotes (G/G)	Number of Heterozygotes (A/G)	Ref Allele/Alt Allele					
			Global	East Asian	South Asian	Europe	American	African
1000G	0/5008	4/5008	A = 0.99920	A = 0.9960	A = 1.000	A = 1.000	A = 1.000	A = 1.000
			G = 0.00080	G = 0.0040	G = 0.000	G = 0.000	G = 0.000	G = 0.000
GnomAD-Genomes	0/140170	7/140170	A = 0.99995	A = 0.9981	A = 1.000	A = 1.000	A = 1.000	A = 1.000
			G = 0.00005	G = 0.0019	G = 0.000	G = 0.000	G = 0.000	G = 0.000
GnomAD-Exomes	0/251194	49/251194	A = 0.999805	A = 0.99904		A = 0.999993	A = 1.000	A = 1.000
			G = 0.000195	G = 0.00096		G = 0.000007	G = 0.000	G = 0.000
ExAC	0/119288	19/119288	A = 0.999841	A = 0.99923		A = 1.000	A = 1.000	A = 1.000
			G = 0.000159	G = 0.00077		G = 0.000	G = 0.000	G = 0.000

### Bioinformatics analyses

**Fig. 3:**
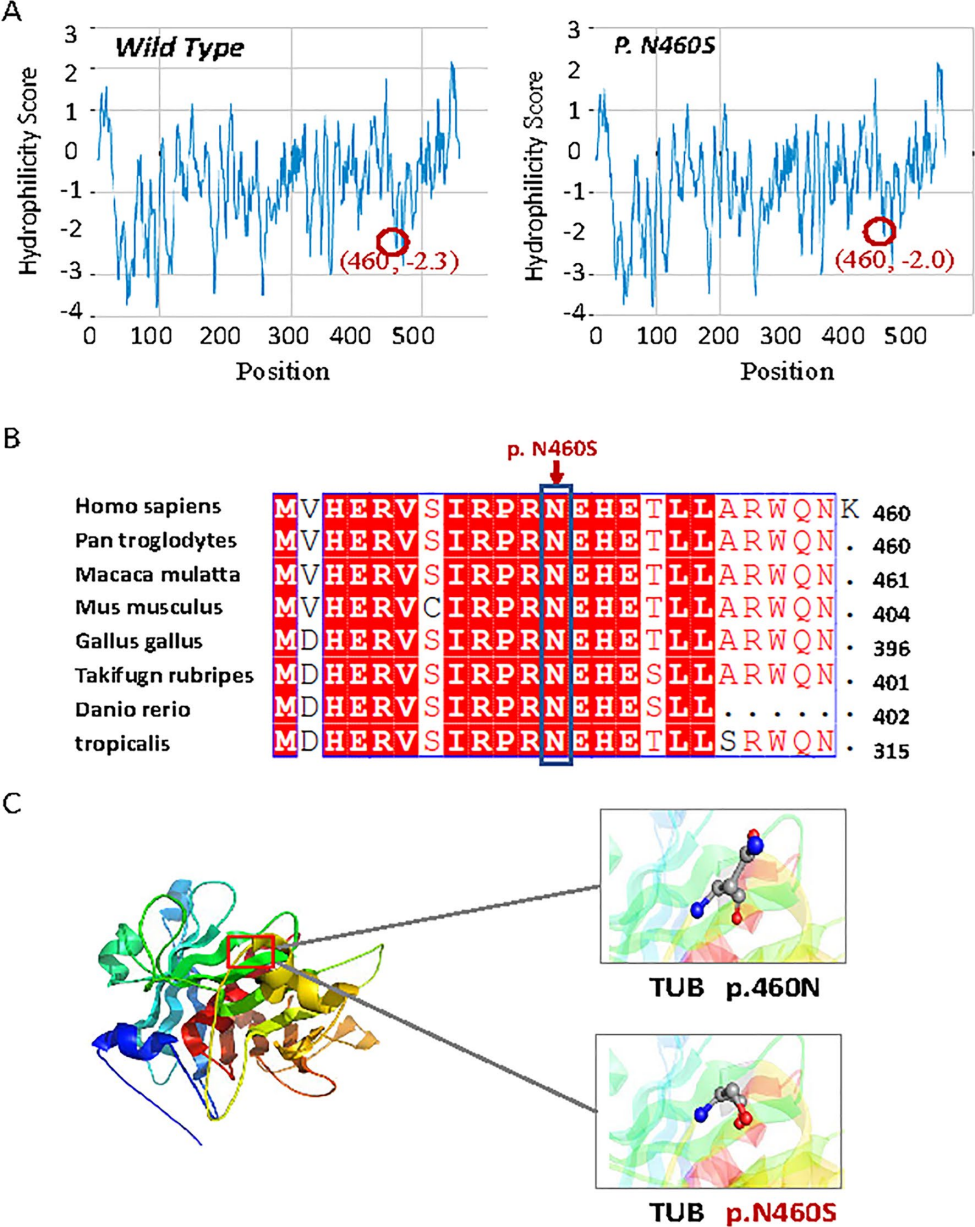
Hydrophobicity analysis of the wild and mutant proteins, multiple alignment of the amino acid sequences around Asn460 in different species, and predicted model structures of the human TUB protein. **A** Hydrophobicity analysis performed by Novopro, indicating a slight decrease in hydrophilicity caused by the variant. **B** Multiple-sequence alignments of TUB around Asn460 among different species, indicating that this variant was highly conserved among species. **C** Predicted structures of the human TUB protein (residues 289 through 561) using PyMOL. The p.Asn460Ser variant in *TUB* is indicated with ball-and-stick models. N, asparagine; S, serine

The effect of the non-synonymous variant in *TUB* (c.1379A > G, p.Asn460Ser) was predicted to be deleterious by PROVEAN with a score of -4.193 (a score ≤ -2.5 is considered deleterious) and damaging by SIFT with a score of 0.035 (a score of 0–0.05 is considered damaging). It was also predicted to be disease-causing by MutationTaster with a score of ~ 1 and damaging by CADD_Phred with a score of 24 (Table 2). The hydrophobicity analysis with regard to the changed residue at codon 460 in TUB indicated a lower hydrophilicity than that of the wild type (Fig. 3A). Phylogenetic analysis indicated that the Asn residue at codon 460 of TUB is highly conserved across diverse species from *tropicalis* to humans (Fig. 3B). Further structural modeling (Fig. 3C) demonstrated that the changed residue at codon 460 of TUB may contribute to an abnormal TUB conformation. According to the ACMG guidelines [23, 24], the c.1379A > G variant in *TUB* could be considered as “likely pathogenic”.

**Table 2:** Functional predictions of the homozygous mutation of TUB in this study

Gene	Accession number	Chromosome position	Exon	Variant Type	Variant	Mutation Taster^a^	PROVEAN^b^ (cutoff = − 2.5)	SIFT^b^ (cutoff = 0.05)	CADD_Phre^c^ (cutoff = 20)
*TUB*	NM_003320.5	11p15.4	11	Missense Variant	c.1379A > G p.Asn460Ser	~ 1	− 4.19	0.035	24
						Disease-causing	Deleterious	Damaging	Damaging

^a^https://www.mutationtaster.org/

^b^http://provean.jcvi.org

^c^http://cadd.gs.washington.edu

### Splicing study of c.1379 A > G variant in *TUB* by minigene assay

**Fig. 4:**
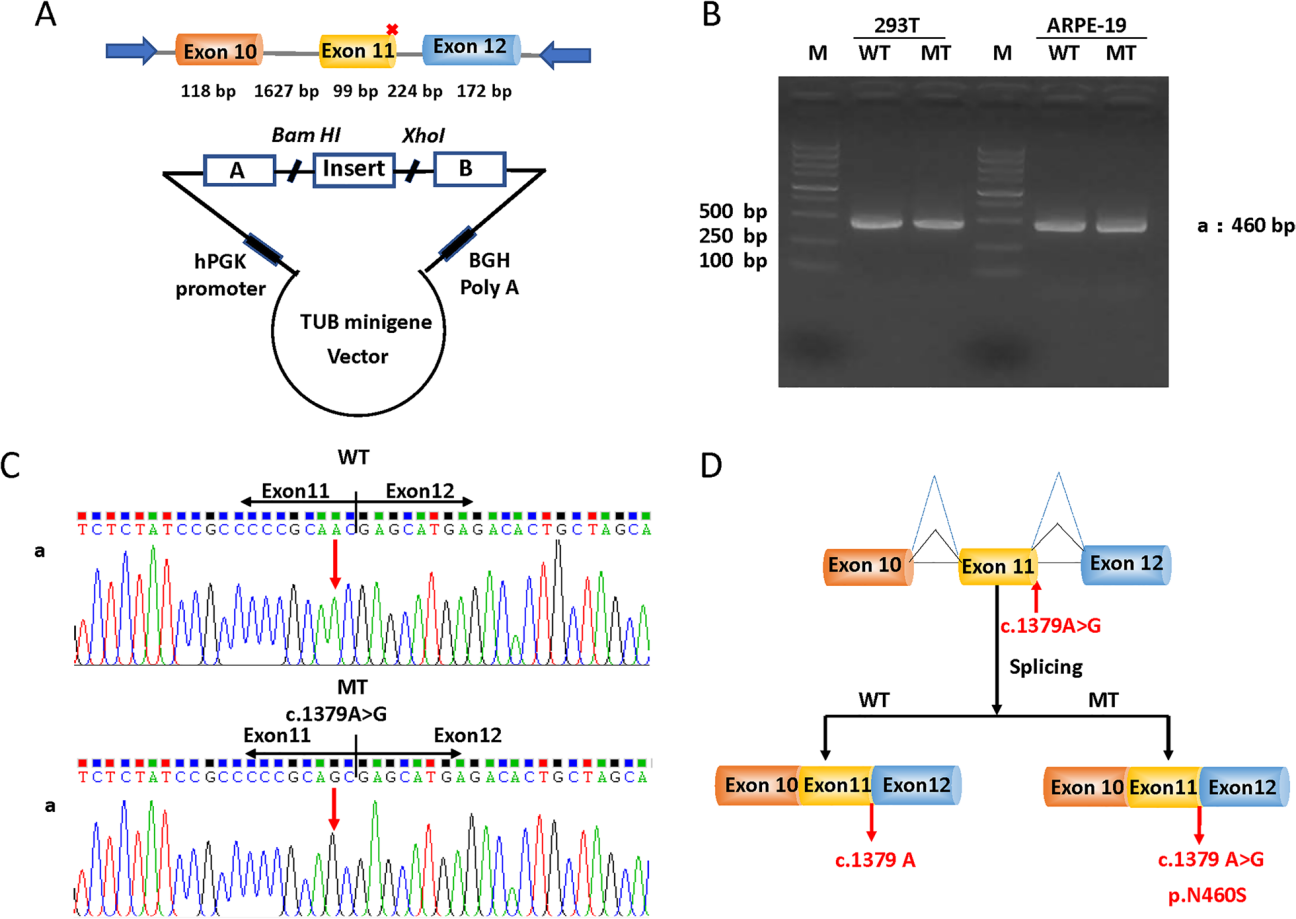
Minigene assay for the c.1379 A > G variant in *TUB.* **A** Schematic illustration of cloned vectors (pMini-CopGFP-TUB minigene), red * indicates the variant site. **B** Electrophoresis of RT-PCR fragments from APRE-19 cells and 293 T cells (original gel is presented in Additional file 1: Fig. S2). **C** Splicing sequences confirmed by Sanger sequencing. **D** Schematic diagram shows no effect of the *Tub* variant (c.1379A > G) on splicing. WT, wild-type; MT, mutant-type

The minigenes pMini-CopGFP-*TUB*-WT/MT (Fig. 4A) were constructed to explore the splicing effect of this variant. The genomic region spanning from Exon10 to Exon12 of *TUB*-WT/*TUB*-MT were cloned into pMini-CopGFP respectively. RT-PCR analysis showed a single band with the expected size of 460 bp for both WT and MT minigenes in two different cell lines (Fig. 4B). And Sanger sequencing confirmed the normal splicing of the minigenes (Exon 10- Exon11- Exon12) (Fig. 4C, D), indicating impact of the variant on splicing could be basically ruled out.

## Discussion

In the current study, we examined a consanguineous family with arRP from central south China and detected a novel homozygous variant (c.1379 G > A: p.Asn460Ser) in *TUB*. This variant co-segregated with the disease in an autosomal recessive manner and was absent in 118 ethnically matched healthy controls. This homozygous variant has not been reported in the literature or deposited in existing databases including gnomAD, 1000G phase3, ExAC, or dbSNP, indicating that this variant is rare. Multiple bioinformatic algorithms predicted that this variant was deleterious or damaging. Conservative analysis showed the codon Asn460 fallen within a highly conserved region across diverse species (Fig. 3B). Further structural modeling showed that amino acid substitution (Ser) of the variant at residue Asn460 could affect the protein conformation, the binding ability of the tubby domain [26], as well as the solubility and stabilization of the mutant protein [27]. Taken together, these data strongly supported the hypothesis that this novel *TUB* variant (c.1379 A > G: p. Asn460Ser) might be the genetic basis of arRP in this Chinese consanguineous family.

For RP-associated diseases in humans, variants in *TUB* are extremely rare. Till now, only one arRP-associated mutation in *TUB* for has been reported in the literature, in which a homozygous frameshift variant resulted in retinal dystrophy, hearing loss, and obesity in a consanguineous UK Caucasian family [12]. Even so, a total of 354 *TUB* variants have been deposited in the ClinVar database with 25 items marked of “pathogenic” or “likely pathogenic” clinical significance, among which at least 3 “pathogenic” or “likely pathogenic” variants have been reported in more than 6 patients with arRP (Additional file 1: Table S3). Interestingly, all these cases mentioned above are generated either by splice-site or protein-truncating mutations in *TUB.* Although the variant found in this study was a single base-substitution, which resulted in a substitution of Asn to Ser at codon 460 in exon 11. According to the ACMG Standards/Guidelines, it’s important to assess the possibility that the variant may act through splicing disruption instead of amino acid change [24]. Considering loss of function is a common mechanism of genetic disease, this homozygous variant coincidently resides on the last but one base of exon 11 of the *TUB* gene, which is classified as a type II splice variant [28]. We first investigate the splicing effect of the variant by RNA analysis from the peripheral blood of the proband as well as healthy controls. But no mRNA of *TUB* was detected in available somatic cells (data not shown), which is consistent with the records in GTEx that the mRNA of *TUB* in peripheral blood is extremely low (Additional file 1: Fig. S1). Considering the unavailability of specific tissues, we further tried to explore the splicing effect of this variant via minigene assay, a powerful way for evaluating the role of novel variants on splicing [29]. The splicing assay in this study, however, showed no differences between the *TUB/WT* and *TUB*/*MT* minigenes in two different cell lines, indicating the possible impact of this variant on splicing could be basically ruled out. A mutant TUB protein induced by the homozygous missense variant instead of loss of function from splicing-error or protein-truncating was probably the pathogenic basis for the simple arRP in this consanguineous family.

To date, five mammalian tubby-like proteins (TULPs) family members (TUB and TULP1 to 4) have been identified with an important role in maintenance of the central nervous system [17]. The TULPs are characterized by a unique “tubby domain” of ~ 260 amino acid residues in the C-terminal [30], which forms a central hydrophobic α-helix traversing the β-barrel structure [11]. The ‘tubby domain’ contains a phosphatidylinositol-binding region specifically binding to phosphatidylinositol 4,5-bisphosphate (PIP2) functioning as membrane-bound transcription regulators [10] and a DNA-binding domain (DBD) that selectively binding to double-stranded DNA [11]. Although the N-terminal motifs of the TULPs are poorly conserved [31], the C-terminal “tubby domains” of the TULPs are remarkedly conserved with 55–95% identity across species from plants to humans [17], implying an evolutionarily conserved function [11]. Structure–function analysis by Sunshin Kim et al. demonstrated that the tubby domain in *TUB* plays a crucial role in its subcellular localization and solubility [26]. In addition, mutations in another *TULP*s family member, *TULP1* (also known as RP14), is known to cause retinal degeneration in both humans and mice [32–35]. Previous studies demonstrated that both *TUB* and *TULP1* are essential for G protein-coupled receptors (GPCRs) mediated trafficking of photoreceptors [30, 36] and MerTK-dependent phagocytosis in retinal pigment epithelium (RPE) and apoptotic cells [37]. Caspase mediated apoptosis and MerTK-dependent phagocytosis are the underlying mechanism of photoreceptor cell death in retinal degeneration [37–40]. Therefore, we presume that the pathogenic missense variant in *TUB* reported here may influence TUB functions through disruption of the ‘tubby domain’, affecting GPCR trafficking, as well as the normal physiology of the photoreceptor cells, and ultimately lead to arRP. Further functional studies, however, are required to elucidate the exact role of *TUB* in arRP. Genetically modified animal models are being developed in the lab to address these points.

Previously reported *TUB-*related RP patients always displayed other systemic abnormalities [12]. Genetic ablation of *Tub* in mice also results in retinal degeneration as well as obesity or hearing deficits in tubby mice [18, 19]. But all these cases previously reported are generated either by splice-site or protein-truncating mutations in *TUB*/*Tub*. In the present study, the patient presented with typical RP without other systemic abnormalities. This can be explained by the following considerations: i) Variant position, the changed residue (p.Asn460Ser) located in the C-terminal tubby domain, which is highly conserved across diverse species from *tropicalis* to humans and completely conserved among the TUB, TULP1, TULP2, and TULP3 family proteins. ii) Variant type, the severity of the phenotype appears to depend on the variant type of the mutant protein, as similar phenotype generated by another *TULP*s family member, *TULP1*. In *TULP1*, RP complicated with Leber congenital amaurosis (LCA) phenotypes were generally induced by splice-site or protein-truncating mutations distributed throughout the gene (Additional file 1: Fig. S3-B), whereas simple RP phenotype was always associated with pathogenic missense mutations located in the tubby domain of *TULP1* (Additional file 1: Fig. S3-A). It’s striking that the *TUB* variant reported here and all missense mutations in *TULP1* described to date, coincidentally disrupted the C-terminal tubby domain with only simple RP phenotype and no other systemic abnormalities.

However, our study has several limitations. First, considering the limits of the WES technique, it is possible that structural variations (SVs), copy number variations (CNVs), mutations in deep intronic or regulatory regions that could induce RP may not be fully detected; Second, some other family members, including both parents and siblings of the proband, are unavailable for further genetic analysis. The issue that whether the novel variant is a de novo or germline mutation inherited from the consanguineous parents is still undefined. In addition, it’s essential to validate the pathogenicity of this variant by large-scale srceenings and functional experiments.

In summary, in this work we reported the identification of a novel rare homozygous variant (c.1379A > G: p.Asn460Ser) of *TUB* in a consanguineous Chinese family with arRP, which was probably the pathogenic basis for arRP in this family. Although the molecular mechanism of the *TUB* gene in arRP remains to be fully elucidated, our study provided valuable insight into the role of *TUB* in arRP, expanded the mutation spectrum of *TUB*, enriched the genotype–phenotype correlation resesrch of arRP, and highlighted the potential role of the *TULPs* family members in retinal function.

## Supplementary Information

**Additional file 1.** Supplementary Figures and Tables.

## Data Availability

The patient data were collected and provided by the Hunan Provincial People’s Hospital, and the NGS and Sanger sequencing data were provided by Berry Genomics. The datasets generated in this study are available from the dbSNP repository (https://www.ncbi.nlm.nih.gov/snp, accession number rs189328202) and the MutationTaster repository (https://www.mutationtaster.org, accession number NM_003320).

## Abbreviations

WES: Whole exome sequencing
arRP: Autosomal recessive retinitis pigmentosa
BCVA: Decimal best-corrected visual acuity
ERG: Axial length and electroretinography
ACMG: The American College of Medical Genetics and Genomics
HGMD: The Human Gene Mutation Database
TULPs: Tubby-like proteins

## References

[1] Chizzolini M, Galan A, Milan E, Sebastiani A, Costagliola C, Parmeggiani F (2011). Good epidemiologic practice in retinitis pigmentosa: from phenotyping to biobanking. Curr Genomics.

[2] Dias M, Joo K, Kemp J, Fialho S, da Silva CAJ, Woo S, Kwon Y (2018). Molecular genetics and emerging therapies for retinitis pigmentosa: basic research and clinical perspectives. Prog Retin Eye Res.

[3] Xu L, Hu L, Ma K, Li J, Jonas J (2006). Prevalence of retinitis pigmentosa in urban and rural adult Chinese: the Beijing Eye Study. Eur J Ophthalmol.

[4] Holt I, Harding A, Petty R, Morgan-Hughes J (1990). A new mitochondrial disease associated with mitochondrial DNA heteroplasmy. Am J Hum Genet.

[5] Goldberg A, Molday R (1996). Defective subunit assembly underlies a digenic form of retinitis pigmentosa linked to mutations in peripherin rds and rom-1. Proc Natl Acad Sci USA.

[6] Nash BM, Wright DC, Grigg JR, Bennetts B, Jamieson RV (2015). Retinal dystrophies, genomic applications in diagnosis and prospects for therapy. Transl Pediatr.

[7] Hartong D, Berson E, Dryja T (2006). Retinitis pigmentosa. Lancet.

[8] Gonzalez-Del Pozo M, Fernandez-Suarez E, Martin-Sanchez M, Bravo-Gil N, Mendez-Vidal C, Rodriguez-de la Rua E, Borrego S, Antinolo G. Unmasking Retinitis Pigmentosa complex cases by a whole genome sequencing algorithm based on open-access tools: hidden recessive inheritance and potential oligogenic variants. J Transl Med 2020, 18(1):73. 10.1186/s12967-020-02258-3 PMC7014749 32050993

[9] Zhang Q (2016). Retinitis pigmentosa. Progress and perspective. Asia Pac J Ophthalmol (Phila).

[10] Santagata S, Boggon T, Baird C, Gomez C, Zhao J, Shan W, Myszka D, Shapiro L (2001). G-protein signaling through tubby proteins. Science.

[11] Boggon T, Shan W, Santagata S, Myers S, Shapiro L (1999). Implication of tubby proteins as transcription factors by structure-based functional analysis. Science (New York, NY).

[12] Borman A, Pearce L, Mackay D, Nagel-Wolfrum K, Davidson A, Henderson R, Garg S, Waseem N, Webster A, Plagnol V (2014). A homozygous mutation in the TUB gene associated with retinal dystrophy and obesity. Hum Mutat.

[13] Ikeda A, Naggert J, Nishina P (2002). Genetic modification of retinal degeneration in tubby mice. Exp Eye Res.

[14] Kleyn P, Fan W, Kovats S, Lee J, Pulido J, Wu Y, Berkemeier L, Misumi D, Holmgren L, Charlat O (1996). Identification and characterization of the mouse obesity gene tubby: a member of a novel gene family. Cell.

[15] Prada P, Quaresma P, Caricilli A, Santos A, Guadagnini D, Morari J, Weissmann L, Ropelle E, Carvalheira J, Velloso L (2013). Tub has a key role in insulin and leptin signaling and action in vivo in hypothalamic nuclei. Diabetes.

[16] Snieder H, Wang X, Shiri-Sverdlov R, van Vliet-Ostaptchouk J, Hofker M, Perks U, Spector T, O'Dell S (2008). TUB is a candidate gene for late-onset obesity in women. Diabetologia.

[17] Ikeda A, Nishina P, Naggert J (2002). The tubby-like proteins, a family with roles in neuronal development and function. J Cell Sci.

[18] Noben-Trauth K, Naggert J, North M, Nishina P (1996). A candidate gene for the mouse mutation tubby. Nature.

[19] Ohlemiller K, Hughes R, Mosinger-Ogilvie J, Speck J, Grosof D, Silverman M (1995). Cochlear and retinal degeneration in the tubby mouse. NeuroReport.

[20] Stubdal H, Lynch C, Moriarty A, Fang Q, Chickering T, Deeds J, Fairchild-Huntress V, Charlat O, Dunmore J, Kleyn P (2000). Targeted deletion of the tub mouse obesity gene reveals that tubby is a loss-of-function mutation. Mol Cel Biol.

[21] Chen S, Tsai Y, Fan S. Drosophila king tubby (ktub) mediates lightinduced rhodopsin endocytosis and retinal degeneration. J Biomed Sci. 2012;19(1). 10.1186/1423-0127-19-101 PMC3541268 23228091

[22] Yao Y, Zheng X, Ge X, Xiu Y, Zhang L, Fang W, Zhao J, Gu F, Zhu Y (2017). Identification of a novel GJA3 mutation in a large Chinese family with congenital cataract using targeted exome sequencing. PLoS ONE.

[23] Miller D, Lee K, Gordon A, Amendola L, Adelman K, Bale S, Chung W, Gollob M, Harrison S, Herman G (2021). Recommendations for reporting of secondary findings in clinical exome and genome sequencing, 2021 update: a policy statement of the American College of Medical Genetics and Genomics (ACMG). Genet Med.

[24] Richards S, Aziz N, Bale S, Bick D, Das S, Gastier-Foster J, Grody W, Hegde M, Lyon E, Spector E (2015). Standards and guidelines for the interpretation of sequence variants: a joint consensus recommendation of the American College of Medical Genetics and Genomics and the Association for Molecular Pathology. Genet Med.

[25] Nielsen M, Lundegaard C, Lund O, Petersen T. CPHmodels-3.0--remote homology modeling using structure-guided sequence profiles. Nucleic Acids Res. 2010, 38:W576–81. 10.1093/nar/gkq535 PMC2896139 20542909

[26] Kim S, Sung H, Lee J, Kim Y, Oh Y, Yoon K, Heo K, Suh P-G (2017). C-terminally mutated tubby protein accumulates in aggresomes. BMB Rep.

[27] Hojgaard C, Kofoed C, Espersen R, Johansson KE, Villa M, Willemoes M, Lindorff-Larsen K, Teilum K, Winther JR (2016). A soluble, folded protein without charged amino acid residues. Biochemistry.

[28] Anna A, Monika G (2018). Splicing mutations in human genetic disorders: examples, detection, and confirmation. J Appl Genet.

[29] Gaildrat P, Killian A, Martins A, Tournier I, Frebourg T, Tosi M (2010). Use of splicing reporter minigene assay to evaluate the effect on splicing of unclassified genetic variants. Methods Mol Biol.

[30] Mukhopadhyay S, Jackson P (2011). The tubby family proteins. Mukhopadhyay and Jackson. Genome Biol.

[31] Wang M, Xu Z, Kong Y (2018). The tubby-like proteins kingdom in animals and plants. Gene.

[32] Lobo G, Au A, Kiser P, Hagstrom S (2016). Involvement of endoplasmic reticulum stress in TULP1 induced retinal degeneration. PLoS ONE.

[33] Woodard D, Xing C, Ganne P, Liang H, Mahindrakar A, Sankurathri C, Hulleman J, Mootha V (2021). A novel homozygous missense mutation p.P388S in TULP1 causes protein instability and retinitis pigmentosa. Mol Vis.

[34] Ullah I, Kabir F, Iqbal M, Gottsch C, Naeem M, Assir M, Khan S, Akram J, Riazuddin SA, Ayyagari R (2016). Pathogenic mutations in TULP1 responsible for retinitis pigmentosa identified in consanguineous familial cases. Mol Vis.

[35] Verbakel S, Fadaie Z, Klevering B, van Genderen M, Feenstra I, Cremers F, Hoyng C, Roosing S (2019). The identification of a RNA splice variant in TULP1 in two siblings with early-onset photoreceptor dystrophy. Mol Genet Genomic Med.

[36] Sun X, Haley J, Bulgakov O, Cai X, McGinnis J, Li T. Tubby is required for trafficking G protein-coupled receptors to neuronal cilia. Cilia. 2012;1(1). 10.1186/2046-2530-1-21 PMC3599646 23351594

[37] Caberoy N, Zhou Y, Li W (2010). Tubby and tubby-like protein 1 are new MerTK ligands for phagocytosis. EMBO J.

[38] Doonan F, Donovan M, Cotter T (2005). Activation of multiple pathways during photoreceptor apoptosis in the rd mouse. Invest Ophthalmol Vis Sci.

[39] Kong L, Li F, Soleman C, Li S, Elias R, Zhou X, Lewis D, McGinnis J, Cao W (2006). Bright cyclic light accelerates photoreceptor cell degeneration in tubby mice. Neurobiol Dis.

[40] Caberoy N, Alvarado G, Li W (2012). Tubby regulates microglial phagocytosis through MerTK. J Neuroimmunol.

