# Supplementary material for: A novel homozygous TUB mutation associated with autosomal recessive retinitis pigmentosa in a consanguineous Chinese family
Source: BMC Med Genomics. 2023 Jan 18;16:9. doi: 10.1186/s12920-023-01430-0 (PMC9847046; doi:10.1186/s12920-023-01430-0)
Supplement: Supplementary file 1 — Additional file 1. Supplementary Figures and Tables. [file 12920_2023_1430_MOESM1_ESM.pdf]

supplemental materials：

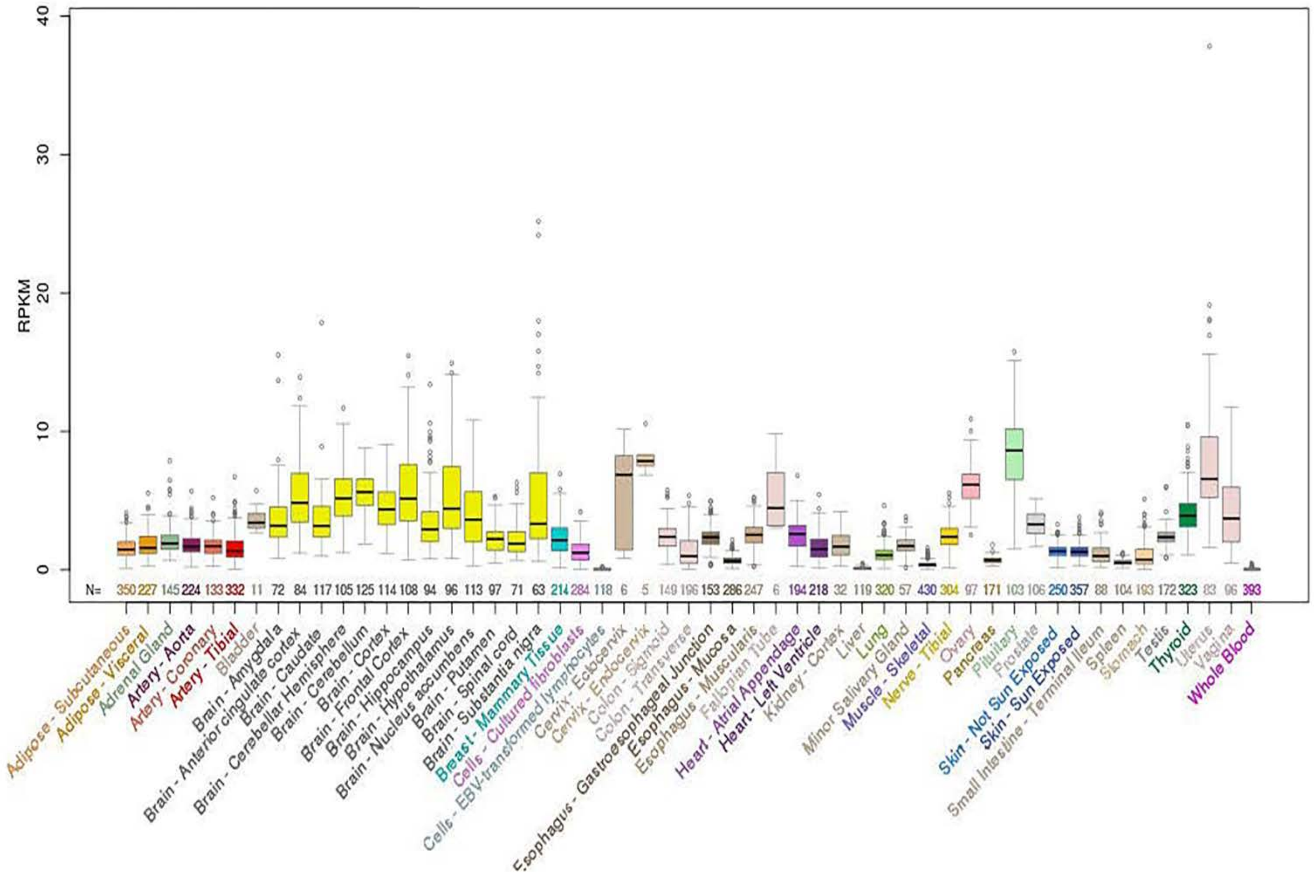

Supplementary Fig. S1. Gene expression of TUB from GTEx (Release V6)
(RPKM: Reads Per Kilobase Million; GTEx: Genotype-Tissue Expression)

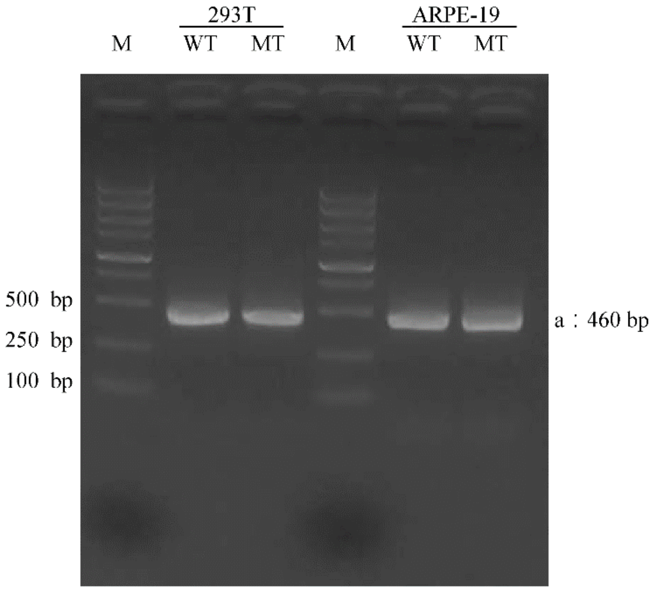

Supplementary Fig. S2. Original gel electrophoresis of RT-PCR fragments from APRE-19 cells and 293T cells

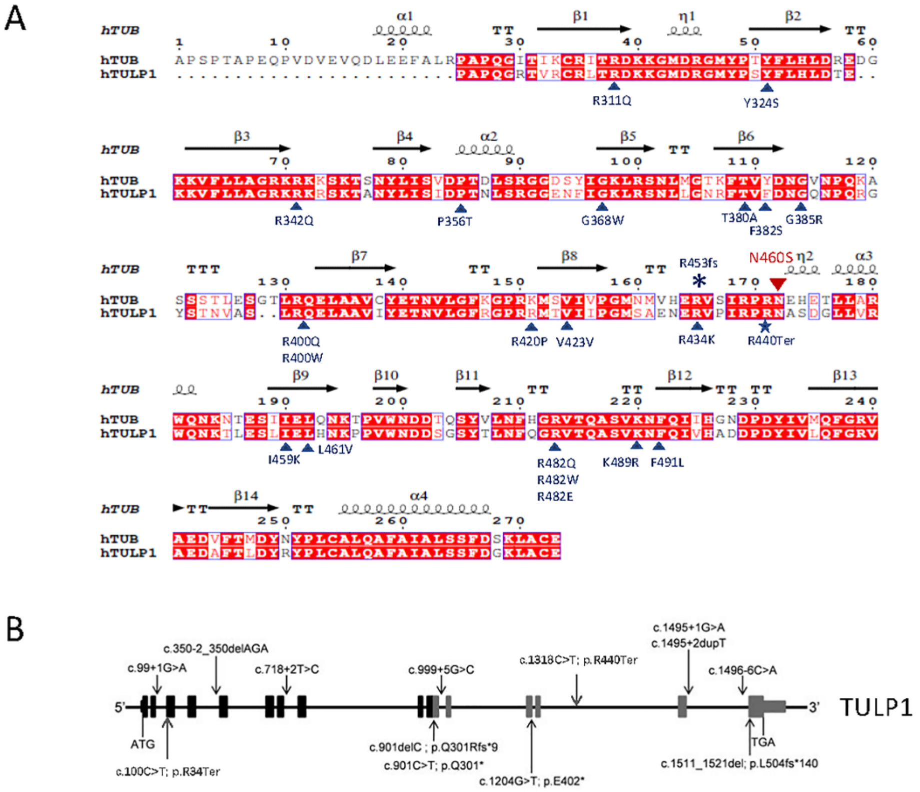

Supplementary Fig. S3. Alignment of the tubby domain of TUB and TULP1 & Splice site and frame shift variants in *TULP1*. (A) Alignment of amino acid sequences of the tubby domain in TUB (NP_003311.2) and TULP1 NP_003313.3). * Represents reported variant residues in TUB protein in the literature; Red rectangle represents missense variant residues reported in current study; Blue rectangle represents pathogenic or likely pathogenic variant residues in TULP1; Blue pentagram represents missense variants in TULP1 in patients with arRP and/or LCA; (B) distribution of splice site variants (above) and nonsense and frame-shift variants (below) in *TULP1* in patients with arRP and/or LCA.

Supplemental Tab.1 List of primers used in the study

Primer Name	Primer Sequence	Tm (C°)	Fragment Size (bp)
TUB-rs189328202	F: 5’-CAAGGATGACGCATAAGAGGA-3’	59	597
	R: 5’-CTTGTTTTGCAGCTCGATGA-3’.		
RAX2-rs559035106	F: 5'-GGT,AGG,TGG,TGA,AGG,TGG,TG-3'	57	240
	R: 5'-CAG,TGG,AGG,GAA,CCG,ATT,T-3'		
RP1-rs367600337	F: 5'-AGG,AAG,GTG,CAG,CCT,GTA,GA-3'	57	224
	R: 5'-AGA,AAT,GCC,TCG,AAG,CTC,TG-3'		
PITPNM3-rs375915718	F: 5'-AGG,CCA,AAA,GCT,GAA,CAG,TG-3'	56	249
	R: 5'-AGT,GGT,GGG,GAA,GCA,AGA,G-3'		

Supplemental Tab. S2 Details of candidate genes detected by WES

Gene Name	Chr	Exon	Gene Bank ID	HGVS (DNA)	HGVS (Protein）	Mutation Type	Functional Predictions				
							Mutation Taster	SIFT_ (cutoff= 0.05)	PROVEAN (cutoff= -2.5)	CADD_ Phred	Polyphen-2 (prob>0.9)
TUB	11	11	NM_003320.4	c.1379A>G (Homozygous）	p.N460S	nonsynomymous SNV	~1 (disease causing)	0.035 (Damaging)	-4.193 (Deleterious）	24	0.178 (Benign）
RAX2	19	2	NM_001319074.2	c.89dup (Hetorozygous)	p.A31Sfs*74	frameshift insertion	~1 (disease causing)	---	---	---	---
RP1	8	2	NM_006269.2	c.424G>A (Hetorozygous)	p.V142I	nonsynomymous SNV	Benign	0.159 （Tolerated）	-0.33 (Neutral)	1.616	0.305 (Benign）
PITPNM3	17	13	NM_031220.4	c.1750C>A (Hetorozygous)	p.V584L	nonsynomymous SNV	Benign	0.002 (Damaging)	-2.66 (Deleterious）	24.5	0.996 (Probably Damaging)

Gene Name	SNP ID	Allele Frequency				Clin Var	Related disease and mode of inheritance
		Mutation Taster	ALFA	ExAC	1000G		
TUB	rs189328202	~1 (disease causing)	0.000113	0.000159	0.0008	Likely pathogenic	Retinal dystrophy and obesity, AR
RAX2	rs559035106	~1 (disease causing)	0.00007	---	0.001	Uncertain significance	Cone-rod dystrophy 11, AD
RP1	rs367600337	Benign	0.00004	---	0	Likely benign	Retinitis pigmentosal 1, AD, AR
PITPNM3	rs375915718	Benign	0	---	---	Uncertain significance	Cone-rod dystrophy 5, AD

Chr: chromosome; The position of variants is determined using the Human Genome38(GRCh38/hg38)；AD:Autosomal Dominant; AR:Autosomal Recessive；Intervar：Clinical Interpretation of genetic variants by ACMG/AMP 2015 guideline. Mutation taster (https://www.mutationtaster.org/), Polyphen-2 (http://genetics.bwh.harvard.edu/pph2/), SIFT (http://sift.jcvi.org), PROVEAN (http://provean.jcvi.org),CADD(http://cadd.gs.washington.edu).

Supplemental Tab.3 Summary of pathogenic/ likely pathogenic variants in the TUB gene associated with RP in the ClinVar database

RefSeqID	Variation ID	Nucleotide variant	Protein variant	Molecular consequence	No. of Submissions	Condition（Inheritance）	Affected status	Clinical significance (Last evaluated)	Reference
NM_177972.3	162490	c.1194_1195del	p.Arg398fs	frameshift	1	Retinal dystrophy and obesity, autosomal recessive	yes	Pathogenic (Mar 01, 2014)	[11]
NM_003320.5		c.1359_1360del	p.Arg453fs		1			Pathogenic /Aug 26, 2019)	not provided
NM_177972.3	804249	c.1215+1G>A	---	splice donor	1	Retinal dystrophy and obesity, autosomal recessive	yes	Likely pathogenic (Aug 26, 2019)	not provided
NM_003320.5		c.1380+1G>A	---		1			Pathogenic (Apr 08, 2021)	
NM_177972.3	865971	c.1387+1G>A	---	splice donor	1	Retinal dystrophy	yes	Likely pathogenic (Jul 12, 2019)	not provided
NM_003320.5		c.1552+1G>A	---		1	not provided	unknown	Uncertain significance (Jul 14, 2021)	
NM_177972.3	1091773	c.1214A>G	p.Asn405Ser	missense	1	Retinitis pigmentosa, autosomal recessive	unknown	Likely benign (Oct 10, 2021)	not provided
NM_003320.5		c.1379A>G	p.Asn460Ser		0		yes	**Likely pathogenic**	**current study**
